# Reliability and validity of the novel self-reported spine functional scale (SSFS) in healthy participants

**DOI:** 10.1186/s13018-021-02620-1

**Published:** 2021-08-25

**Authors:** Wei Li, Jie Ding, Xiujuan Hao, Wenjun Jiang, Hongqiang Song, Yanming Zhang, Yan Tan

**Affiliations:** 1 National Institute of Sports Medicine of China, Beijing, China; 2 Changzhi University, Changzhi, China; 3 Shandong First Medical University – Tai’an Campus, Sports Medicine, Taian, Shandong China; 4 Shandong First Medical University, Sports Medicine, Jinan, Shandong China; 5 The Second Affiliated Hospital of Shandong First Medical University, Taian, China; 6 Nanjing University of Posts and Telecommunications, Sports Department, Nanjing, China

**Keywords:** Spinal self-function assessment, Reliability, Validity, BMI

## Abstract

**Objectives:**

To develop the novel self-reported spine functional scale (SSFS) and conduct reliability and validity analysis, so that the public can better understand their own spine function in a more simple and scientific way, so as to effectively prevent spinal disorders and improve the quality of life through targeted rehabilitation therapeutic measures.

**Methods:**

This study was approved by an institutional review board, and all subjects gave informed consent to participate.

**Results:**

(1) Using Spearman correlation analysis to evaluate the content validity, each item was significantly correlated with the total score, and the project design was reasonable. The exploratory factor analysis method is used to evaluate the structural validity of the scale, and the standing position and the lying position of the posture evaluation can be attributed to the factor 2, which is called posture evaluation: the cervical flexor strength, the flat support, the prone back, and the supine knee. The back arch of the wall and the angel on the wall is attributed to factor 1, called the overall spine function test, and the cumulative contribution rate of the two factors was 46.057%. Confirmatory factor analysis showed that the two-factor model fits well (*χ*^2^/df = 2.440, RMSEA = 0.04 < 0.05, GFI = 0.945, AGFI = 0.920, CFI = 0.967, IFI = 0.967, TLI = 0.951, GFI, AGFI, CFI, IFI, and TLI are > 0.90) and the validity is ideal. (2) The test-retest reliability shows that the test-retest reliability of each entry, each dimension, and the total score is greater than 0.5, and the test-retest reliability is high. The Cronbach *α* coefficient was used to evaluate the overall internal consistency of the scale, *α* > 0.70, indicating that the scale has high reliability. After deleting each item one by one, the *α* coefficient is 0.692–0.717, and there is no significant increase. (3) Sex and occupation did not affect the level of spinal function (*P* > 0.05), and there was interaction. Different BMI levels significantly affected the score of spinal function (*P* < 0.05). The rate of spinal dysfunction in overweight and obese subjects was significantly higher than the normal group; the overall score of spinal function was worse than the normal group.

**Discussion:**

The reliability and validity analyses of this study verified the reliability and scientificity of SSFS in the young healthy population. Body weight had a significant influence on SSFS score, and the performance levels were different for the two sexes.

**Conclusion:**

The novel Self-Reported Spine Functional Scale (SSFS) has high reliability and validity and is applicable to the self-assessment and maintenance of spinal health and the prevention of related spinal disorders in the young healthy population. Body weight has a significant influence on the SSFS score in healthy young people. Overweight and obese males were found to be more likely to have spinal dysfunction, while underweight males displayed poor cervical flexor muscle strength. Underweight females were found to have better overall spinal function and stronger cervical flexor muscle strength.

**Supplementary Information:**

The online version contains supplementary material available at 10.1186/s13018-021-02620-1.

## Introduction

Spine health has an inseparable influence on health-related quality of life. According to recent statistics, about two thirds of the postsecondary students in China suffer from cervical spondylosis, and a significant number suffer from lumbar spondylosis [1]. Since the rising incidences of spine-related health concerns among healthy and young population, it is also becoming a major public health issue imposing great social and economic burden on individuals and societies worldwide, seriously influencing public health [2, 3]. Effective preventive measures are a key to address the issue, but the general public’s inadequate level of medical knowledge and the inaccessibility of healthcare facilities to respond to the clinical needs cause people not to accurately recognize their own spinal health condition, also unable to timely prevent the occurrence of the spinal dysfunction or disorders through modern rehabilitation science. This lack of connection and application between basic medical sciences and clinical medicine also occurs in other countries. Therefore, in the recent years, translational medicine based on patient participation has emerged in the European and American countries [4]. This not only strengthens the connection between basic scientific research and clinical practice, reduces the medical burden, but also provides accessibility and convenience for public health. Therefore, it is necessary to provide a self-assessment spinal function evaluation tool for the general public.

The purpose of this study is to develop the novel self-reported spine functional scale (SSFS) and conduct reliability and validity analysis, so that the public can better understand their own spine function in a more simple and scientific way, so as to effectively prevent spinal disorders and improve the quality of life through targeted rehabilitation therapeutic measures.

## Methods

### Data collection

From September 2018 to February 2019, 916 healthy young adults were randomly selected to self-evaluate their spine function using the novel spinal function assessment scale. The sample consisted of 752 males and 164 females, composed of regular office workers, postsecondary students and air force recruits. Two weeks after initial data collection, 173 subjects (consisted of 68 males and 105 females) from the sample population were randomly selected again for test-retest reliability analysis of the scale.

#### Inclusion criteria

The inclusion criteria are as follows: healthy young adults aged between 18 and 35, inclusive; with no previous history of spinal surgery; no major trauma or injuries to the spine; no genetic spinal disorders; and no clinical signs of pain or discomfort during the testing period.

#### Exclusion criteria

The exclusion criteria are as follows: severe cognitive impairment; inability to communicate through writing or speech; severe psychiatric disorders; and severe underlying illnesses that prevented completion of the study.

### Methodology

#### Construction of the SSFS scale

By summarizing the knowledge related to orthopedic measurement and motor function evaluation, this study drew on, improved, and innovated functional testing methods for the spine and divided the assessment of spinal function into three categories: postural assessment, muscle strength testing, and overall functional assessment. Then, the Delphi expert consultation method was used to establish the factors and items of the scale through two rounds of expert consultation. A preliminary framework for the spinal function assessment scale was established, incorporating eight influencing items forming the item pool (Additional file 1). It includes postural assessment in standing and supine; muscle strength tests of neck flexors and abdominal core muscles; and functional assessment of prone press-up, supine knee-to-chest, wall roll-down, and wall angel. Each entry is graded on a 3-point scale. A score of 0 indicates loss of spinal motor function, a score of 1 indicates severe spinal motor dysfunction, a score of 2 indicates mild to moderate spinal motor dysfunction, and a score of 3 indicates good spinal motor function. The maximum score that can be achieved is 24. A higher score indicates better spinal function: total score of 20–24 means good, 15–19 means satisfactory, and 0–14 means poor or unsatisfactory [5, 6].

#### Testing method

All subjects were marked by showing as much of the torso as possible, male subjects wore shorts, and female subjects wore fitted clothing. Then, each subject self-administered the items on the spine functional self-assessment scale by reading the instructions (Additional file 2) or listening to a professional’s verbal instructions, with necessary monitoring and guidance from the researcher during the completing of the scale. Two weeks after initial testing, a randomly selected 173 people completed the self-assessment scale again under supervision of the same group of researchers for the analysis of test-retest reliability, Spearman correlation analysis was performed.

#### Data processing

Statistical analysis was performed using SPSS22.0 and AMOS21.0 statistical software, with *P* < 0.05 representing statistically significant difference, and continuous variable was expressed as mean ± standard deviation, while discrete variable was expressed in percentage. The items of the scale were scored as single-item ordered data, and Spearman’s correlation was used to calculate the correlation coefficients between each item and the total score, and items with low correlation (*r* < 0.4) or correlation not reaching significance with the total score (*P* > 0.05) were removed to evaluate the content validity of the scale. Exploratory factor analysis, confirmatory factor analysis, and average variance extracted analysis (AVE) were used to evaluate the structural and discriminant validity of the scale. Internal consistency and test-retest reliability analysis were completed to evaluate the reliability of the scale. Internal consistency was assessed using Cronbach’s alpha (*α*) coefficient, and the contribution of each item to the internal consistency of the scale was evaluated by the change in Cronbach's *α* coefficient after each item deletion. A multifactorial ANOVA was conducted to analyze the effect of sex, occupation, and BMI levels on the SSFS score, and the chi-squared test was used for comparisons of poor spinal function rates.

## Results

### General

Valid survey was conducted on 916 subjects (752 males and 164 females) with a mean age of 21.16 ± 8.67 years. From the initial sample, 173 subjects consisting of 68 males and 105 females were randomly selected for a 2-week re-evaluation using the SSFS. The basic profile of the subjects is shown in Table 1.

**Table 1:** Basic profile of the subjects ($\overline{\mathrm{x}}$±s)

Subjects	Age (years)	Height (cm)	Bodyweight (kg)
**All subjects(*n*= 916)**	**21.16 ± 8.67**	**171.62 ± 8.84**	**65.67 ± 21.17**
**Retesting subjects(*n*= 173)**	**25.91 ± 8.67**	**166.99 ± 8.16**	**59.36 ± 11.59**

#### Results of expert consultation and analysis of the scale items

Ten domestic experts with professional and academic background in the field of sports medicine and sports rehabilitation were invited for this study, all of them have senior titles. The expert consultation form was distributed twice, and the response rate was 100% during both times, indicating high motivation towards the study [7]; the coefficient of expert authority (Cr) [8] was 0.9, representing high expert authority. All eight items of the scale were retained based on expert opinion.

### Validity analysis

#### Content validity

Spearman correlation analysis was used to evaluate the correlation between each item and the total score. The results showed that the entries were significantly correlated with the total score; therefore, no item was deleted from SSFS (Table 2).

**Table 2:** Analysis of the correlation between each item and the total score

Item	***r***
**Standing postural assessment**	0.399**
**Supine postural assessment**	0.427**
**Neck flexor muscle strength**	0.571**
**Abdominal core muscle strength**	0.620**
**Prone press-up**	0.558**
**Supine knee-to-chest**	0.560**
**Wall roll-down**	0.602**
**Wall angel**	0.519**

*r*: correlation coefficient of each entry with the total score, **P < 0.01

#### Structural validity

##### Exploratory factor analysis

**Fig. 1:**
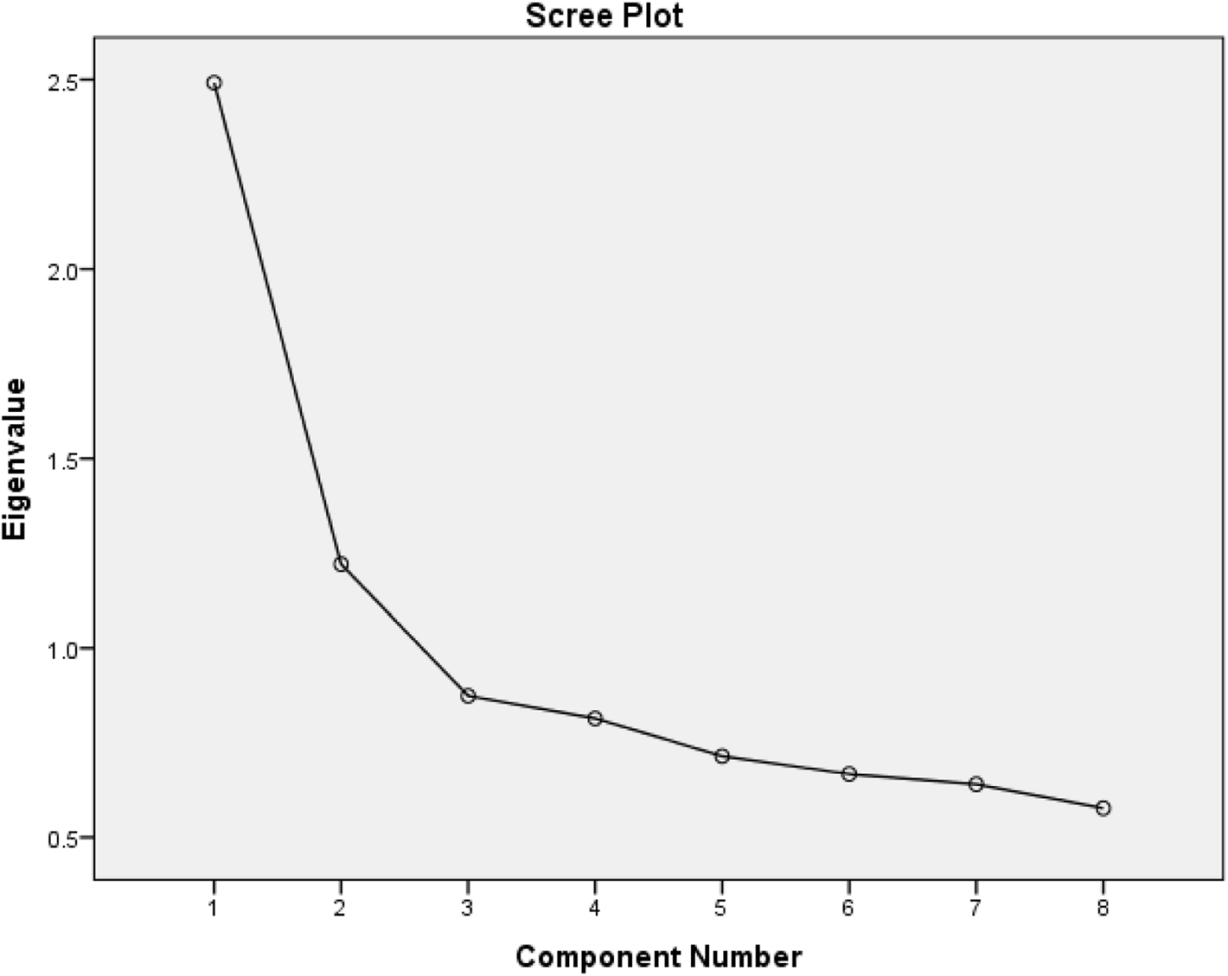
Scree plot of factor characteristics

Exploratory factor analysis was used to evaluate the structural validity of the scale. In this study, the KMO value was 0.779 and the Bartlett’s test of Sphericity resulted a value of 829.680, and *p* < 0.01, indicating that the source data was suitable for factor analysis. The two metric factors with eigenvalues > 1 were extracted, and the contribution rate of factor 1 was 30.900%, the contribution rate of factor 2 was 15.156%, and the cumulative contribution rate was 46.057%. Figure 1 shows the scree plot of the factor characteristics. According to the scree plot, there is a steep slope from components 1 to 3 and then the leveling off after component 3, indicating that it is appropriate to extract two metric factors from the eight components.

Factor loading matrices were obtained using varimax orthogonal rotation, as detailed in Table 3, with all entries retained for subsequent analysis. The standing and supine postural assessment can be attributed to factor 2, referred to as the postural assessment; neck flexor muscle strength, abdominal core muscle strength, prone press-up, supine knee-to-chest, wall roll-down, and wall angel can be attributed to factor 1, which was referred to as the overall functional test.

**Table 3:** Factor loading for the SSFS scale

Item	Factor loading	
	1	2
**Standing postural assessment**	**0.385**	**0.680**
**Supine postural assessment**	**0.378**	**0.719**
**Cervical flexor muscle strength**	**0.642**	**− 0.235**
**Abdominal core muscle strength**	**0.597**	**− 0.242**
**Prone press-up**	**0.595**	**0.137**
**Supine knee-to-chest**	**0.625**	**− 0.222**
**Wall roll-down**	**0.620**	**− 0.138**
**Wall angel**	**0.533**	**− 0.179**

##### Confirmatory factor analysis

Confirmatory factor analysis was performed on the overall fitting of the two-factor model of the SSFS using AMOS 21.0. The initial model resulted in the following values (Table 4): *χ*^2^/df (chi-squared over degree of freedom) = 2.440, RMSEA (root mean square error of approximation) = 0.04 < 0.05, GFI (goodness of fit index) = 0.945, AGFI (adjusted goodness of fit) = 0.920, CFI (comparative fit index) = 0.967, IFI (incremental fit index) = 0.951, TLI (Tucker-Lewis index) = 0.951. GFI, AGFI, CFI, IFI, and TLI were all > 0.90.

**Table 4:** The overall fit indices of SSFS

*χ*^2^/df	RMSEA	GFI	AGFI	CFI	IFI	TLI
2.440	0.04	0.945	0.920	0.967	0.967	0.951

A1 standing postural assessment; A2 supine postural assessment; B1 neck flexor muscle strength; B2 abdominal core muscle strength; B3 prone press-up; B4 supine knee-to-chest; B5 wall roll-down; B6 wall angel

**Fig. 2:**
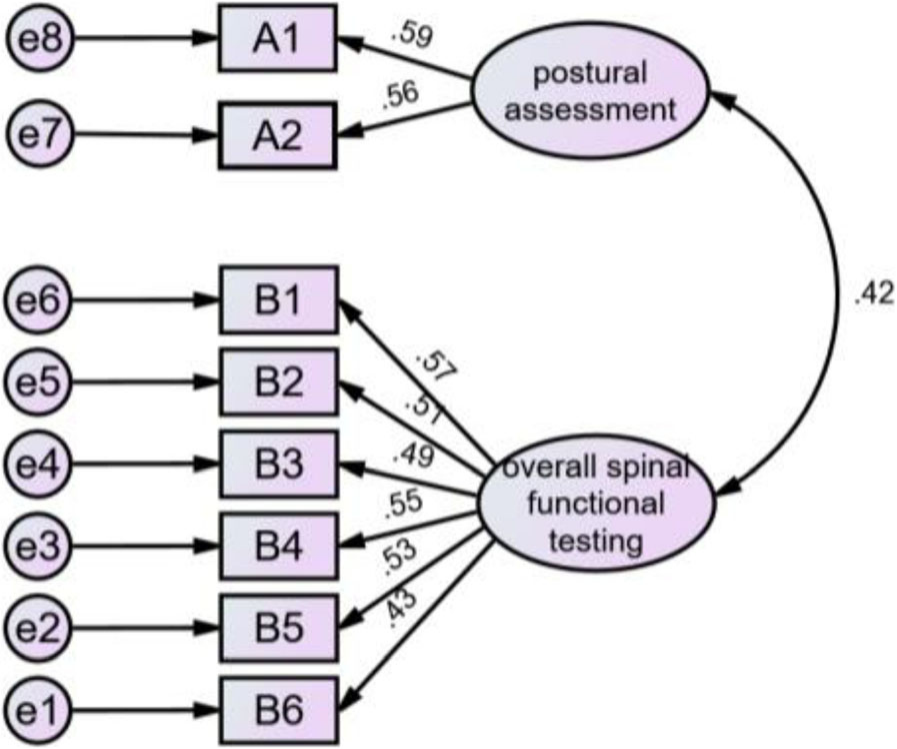
The standardized parameter model diagram of SSFS. A1 standing postural assessment; A2 supine postural assessment; B1 neck flexor muscle strength; B2 abdominal core muscle strength; B3 prone press-up; B4 supine knee-to-chest; B5 wall roll-down; B6 wall angel

In summary, the SSFS model fitted ideally with the standardized parameter path diagrams shown in Fig. 2. Factor loadings were statistically significant in all cases: Score was between 0.56 and 0.59 for postural assessment and was between 0.43 and 0.57 for the overall spinal functional testing.

#### Discriminant validity

The AVE evaluation variance extraction was used to analyze the discriminant validity of the SSFS, as shown in Table 5.

**Table 5:** Discriminant validity analysis of SSFS

	Postural assessment	Spinal function
Postural assessment	0.2659	
Overall spinal function	0.416**	0.331
Square root of AVE	0.516	0.575

**P < 0.01; the diagonal is the AVE evaluation variance extractions

The correlation between postural assessment and overall spinal function was significant (*P* < 0.01), and the absolute values of the correlation coefficients were less than 0.5 and less than the square root of the corresponding AVE, suggesting that the latent variables are correlated with each other and distinguishable from each other. This in turn signifies the discriminant validity of the scale data is ideal.

### Reliability analysis

#### Internal consistency

The overall internal consistency of the scale was evaluated using the Cronbach’s *α* coefficient (Table 6) which showed *α* = 0.727. *α* > 0.70 indicates that the scale has high reliability. After removing each individual item, the *α* coefficients maintained between 0.692 and 0.717, with no significant change.

**Table 6:** Reliability analysis of SSFS

Item	Cronbach’s *α* coefficient after removal of each item
Standing postural assessment	0.717
Supine postural assessment	0.717
Neck flexor muscle strength	0.699
Abdominal Core muscle strength	0.692
Prone press-up	0.699
Supine knee-to-chest	0.702
Wall roll-down	0.698
Wall angel	0.701

#### Test-retest reliability

Spearman’s correlation coefficient was used to evaluate the test-retest reliability of the scale. The results showed high test-retest reliability for each item, for each factor, as well as for the total score, *r* > 0.5, which signifies high test-retest reliability of the SSFS (Table 7).

**Table 7:** Test-retest reliability analysis of SSFS using Spearman correlation coefficients

Factor	Item	Test-retest confidence level of each entry	Retesting confidence of each factor	Overall retest confidence
Postural assessment	Standing postural assessment	0.639**	0.800**	0.914**
	Supine postural assessment	0.567**		
Overall functional assessment	Neck flexor muscle strength	0.739**	0.889**	
	Abdominal core muscle strength	0.873**		
	Prone press-up	0.544**		
	Supine knee-to-chest	0.880**		
	Wall roll-down	0.942**		
	Wall angel	0.505**		

*r*: correlation coefficient of each item with the total score, **P < 0.01

### Influence of BMI levels on spinal function

A total of 819 subjects (consisted of 681 males and 138 females) remained after screening out the subjects with incomplete occupation information. Six hundred eighteen of whom were military (air force) recruits, 78 were postsecondary students, and 123 were other regular workers. According to the Guideline for the Prevention and Control of Overweight and Obesity in Chinese Adults (2006), BMI ≥ 28 kg/m^2^ is considered obese, 24 ≤ BMI < 28 kg/m^2^ is considered overweight, BMI < 18.5 kg/m^2^ is considered underweight, 18.5 ≤ BMI < 24 kg/m^2^ is considered appropriate weight [9, 10]. Table 8 shows SSFS scores of subjects within each BMI level. A low SSFS score indicates poor spinal function, where a total score of 20–24 means good spinal function, 15–19 means satisfactory spinal function, and 0–14 means poor or unsatisfactory spinal function.

**Table 8:** Spinal function in subjects with different BMI levels

Sex	Group	Spinal function				Total	Percentage of poor spinal function
			Good	Satisfactory	Poor		
Male	BMI level	Underweight	11	10	2	23	8.70%
		Normal	271	197	13	481	2.70%
		Overweight or obese	79	87	11	177	6.21%
	Total		361	294	26	681	3.82%
	*χ*^2^						11.144
	*P*						0.025
Female	BMI level	Underweight	21	15	2	38	5.26%
		Normal	29	33	32	94	34.04%
		Overweight or obesity	0	3	3	6	50%
	Total		51	51	36	138	26.09%
	*χ*^2^						16.082
	*P*						0.003

As shown in Table 8, the rates of poor spinal function in the underweight and overweight or obese groups of male subjects were significantly higher compared with the normal BMI group (*P* < 0.05). The rates of poor spinal function in the overweight or obese groups of female subjects were significantly higher than that in the normal group (*P* < 0.05).

Table 9 and Table 10 respectively display the individual SSFS item scores as well as overall scores of the male and female subjects separated by BMI levels. The spinal function of each group was compared using one-way ANOVA. Although the scores of postural assessment in standing, neck flexor muscle strength, and wall angel were not significantly different among the three BMI groups, the overall spinal function of the overweight and obese group in the male subjects was significantly worse than that of the normal group (*P* < 0.05). Only the neck flexor muscle strength was significantly lower in the male underweight group than that in the normal group (Table 9).

**Table 9:** Spinal function in male subjects with different BMI levels

Item	Underweight group (*n* = 23)	Normal group (*n* = 481)	Overweight or obese group (*n* = 177)
Standing postural assessment	2.52 ± 0.59	2.53 ± 0.55	2.48 ± 0.62
Supine postural assessment	2.57 ± 0.66	2.52 ± 0.60	2.40 ± 0.72*
Neck flexor muscle strength	2.30 ± 0.82	2.59 ± 0.59#	2.56 ± 0.62
Abdominal core muscle strength	2.04 ± 0.88	2.27 ± 0.79	2.08 ± 0.83*
Prone press-up	2.17 ± 0.83	2.32 ± 0.63	2.17 ± 0.76*
Supine knee-to-chest	2.48 ± 0.51	2.55 ± 0.62	2.40 ± 0.68*
Wall roll-down	2.13 ± 0.81	2.37 ± 0.64	2.25 ± 0.72*
Wall angel	2.61 ± 0.58	2.44 ± 0.67	2.40 ± 0.70
Total	18.83 ± 3.33	19.59 ± 2.83	18.74 ± 3.05*

^#^Significantly different compared to the overweight or obese group, *P* < 0.05. *Significantly different from the normal group, *P* < 0.05

**Table 10:** Spinal function in female subjects with different BMI levels

Items	Underweight group (*n* = 38)	Normal group (*n* = 94)	Overweight or obese group (*n* = 6)
Standing postural assessment	2.42 ± 0.76	2.28 ± 0.74	1.83 ± 0.98
Supine postural assessment	2.47 ± 0.60	2.33 ± 0.69	1.83 ± 0.98#
Neck flexor muscle strength	2.55 ± 0.72	2.18 ± 0.84#	1.67 ± 0.82#
Abdominal core muscle strength	1.76 ± 1.17	1.54 ± 1.10	1.17 ± 0.98
Prone press-up	2.82 ± 5.11	1.74 ± 0.90#	1.00 ± 1.26
Supine knee-to-chest	2.50 ± 0.73	2.27 ± 0.86	2.00 ± 0.00#
Wall roll-down	2.40 ± 0.72	2.04 ± 0.85	2.00 ± 0.00#
Wall angel	2.82 ± 0.46	2.44 ± 0.76#	2.33 ± 0.82
Total	18.97 ± 3.55	16.82 ± 4.02#	14.00 ± 3.63#

^#^Significantly different compared to the underweight group, *P* < 0.05

In female subjects, there was no significant difference between the overweight or obese group and the normal group in the overall spinal function, but there was a tendency for the scores to be lower than that of the normal group. In the overweight and obese groups, the postural assessment in supine, neck flexor muscle strength, supine knee-to-chest, wall roll-down, and the total score were significantly lower than those items in the underweight group (*P* < 0.05). For the other items, there was a tendency for the scores of the overweight or obese group to be lower compared with the underweight group, despite the differences not reaching statistical significance. Neck flexor muscle strength, prone press-up, wall angel, and the total score of the normal group were significantly lower than the underweight group (see Table 10).

## Discussion

Currently, the development and application of the functional assessment scales are mainly aimed at specific clinical condition or pathology [8, 11, 12]. The evaluation process usually requires medical professionals to complete, and the scales are unable to provide clinical evidence of the disease prevention for the healthy population. Garcia et al. [13] developed a novel shoulder functional movement test for the long-term clinical follow-up of patients with shoulder pain and verified the reliability and validity of the self-assessment test. It provided new ideas for functional self-assessment but also cannot be applied to injury prevention in the healthy population because of the limitations of its clinical application. In terms of spinal function evaluation, most of the currently existing questionnaire-based self-evaluation scales for cervical and lumbar spine disorders, such as Oswestry Disability Index (ODI) for low back pain and Neck Disability Index (NDI) for the cervical spine which are recommended by the American Physical Therapy Association [14, 15], are only applicable to clinical pathological conditions and lack a “holistic view” of the healthy spinal function. Gabel et al. [16] developed a questionnaire-based clinical evaluation of the overall spinal function; however, it did not contain an evaluation of functional positioning (e.g., cervical, thoracic, and lumbar segments of the spine) and the functional properties (e.g., posture, flexibility, stability, etc.). The Self-Reported Spine Functional Scale (SSFS) was developed based on modern medical rehabilitation concepts and the principles of “integrative, comprehensive, accurate and practical,” in an attempt to provide the public with a simple, reliable, and user-friendly tool for the self-assessment of spinal function in the healthy population. This can effectively and timely prevent the occurrence of spine-related disorders and to promote the awareness of spinal function and to motivate evidence-based actions to maintain healthy spinal functions.

### Construction of the SSFS

Studies have shown that a large percentage of the postsecondary students suffer from spinal dysfunction, and regular exercise can improve the spine flexibility and stability [1]. The assessment of spinal condition is composed of three major components: posture assessment, spinal muscle strength assessment, and function [17, 18]. Combining the concepts of scientific, comprehensive, simple, and reproducible, this study adapted from the conventional assessment methods, improved and innovated to develop a novel Self-Reported Spine Functional Scale (SSFS) containing 8 assessment items.

In the postural assessment component, positioning of the cervical spine, thoracic spine, and lumbar spine on the three-dimensional plane was evaluated. The spine is the central axial structure that maintains the human posture; therefore, the evaluation of human posture with the body surface markers can simply and accurately reflect the functional state of the spine. Many studies have suggested that different body positions (such as in standing, in lying, or in long-sitting) will cause spine and different muscle groups to activate differently [19, 20], so this study incorporated the innovative qualitative assessment of lumbar spine posture in side-lying, with an aim to evaluate the position of spine with the effect of gravity in the coronary plane. It is also a reflection of flexibility and stability of the lumbar spine and bilateral symmetry. In addition, the standing posture assessment is a practical and relatively accurate method to evaluate the lumbar spine and pelvis positioning in the sagittal plane, which is widely used in modern rehabilitation practice. The remaining test items are common orthopedic measurement methods [21].

The muscle strength test includes assessment of the neck flexor muscle strength and abdominal core muscle strength. Paraspinal musculatures are the key structures which function to maintain the stability of the spine and help with the coordinated movement; therefore, relevant tests are essential in the evaluation of spinal function. In view of the special anatomical characteristics of the cervical spine and the high incidence of related diseases [3], cervical flexion was selected to represent cervical motor function [16]. Planking was used to determine abdominal core muscle strength and evaluate the overall spinal muscle strength, stability and movement coordination [22]. In order to facilitate the self-evaluation procedure, the testing methods and grading standards of the two tests were reasonably adapted.

The global functional test is used to evaluate the functional movement of the spine. Prone press-up and supine knee-to-chest respectively evaluate the flexibility and coordination of spine extension and flexion, which are practical assessment methods adapted from the McKenzie method of assessment and management for spinal disorders [23]. Wall roll-down is included to evaluate the flexibility and coordination of the overall movement of the spine, and especially the mobility, stability and flexibility of each segment of the spine, and is an innovative functional testing method adapted from the Slump Test [21]. The wall angel was used to evaluate the posture and mobility of the thoracic spine in the sagittal plane, flexibility of the upper extremities, and upper spinal movement coordination [17].

Therefore, the assessment items on the SSFS are all supported by literature and modern rehabilitation concept and strictly define the evaluation methods and grading standards. SSFS in turn can accurately and comprehensively portray the overall functional mobility of the spine. The scale can also be easily interpreted, completed by subjects with high reproducibility and effective application. SSFS has undergone expert consultation and Spearman correlation analysis, where all of the above 8 items on the SSFS significantly correlated with the total score and had been retained [24].

### Reliability and validity analysis of SSFS

The initial sample population of the study was composed of 917 healthy young adults. Compared with similar studies, the sample size of this study was larger, and the age range of the subject population was narrower (between 18 and 30 years old), with occupation mainly consisted of air force recruits and postsecondary students. The collection of relevant data has high statistical importance for the reliability and validity analysis of SSFS. The expert consultation evaluation signifies that the experts in this study have high academic and professional authority, motivated participation, and reasonable and reliable consultation results.

### Validity analysis

Validity refers to whether the scale can accurately and effectively measure the corresponding characteristics. The higher the validity, the more effective the scale is, and the more it reflects correct test results. Validity is generally analyzed based on three aspects: content validity, structure validity, and discriminative validity.

Content validity refers to the conformity between the elements of the scale and what the scale is intended to measure, namely the applicability and representation of the items in the scale [25]. In this study, domestic experts in the field of sports medicine and sports rehabilitation were consulted for the selection of scale assessment items. Spearman correlation analysis was used to evaluate the correlation between each item on the scale and the total score. All items of the scale were kept, and statistical analysis confirmed the reasonable design of the items.

Structural validity, also known as construct validity, indicates whether the structure of the scale is consistent with the theoretical assumption of the scale formulation and whether the components of the measurement results are consistent with the purpose the researcher intends to measure. The use of factor analysis to evaluate the structural validity of a scale is a relatively well-accepted method [8]. In this study, exploratory factor analysis and confirmatory factor analysis were conducted. In exploratory factor analysis, the factor loading matrix divided the eight items in the scale into two factors with a cumulative contribution rate of 46.057%. The factors were named the postural assessment factor and overall spinal function factor, establishing the two factors of the scale [26]. The neck flexor and abdominal core muscle strength tests and the spine functional test were categorized into the same factor, which is basically in line with the design of this scale.

Confirmatory factor analysis was conducted to explore the consistency of the factor structure of the scale with the collected data and whether each item in the scale can be effectively used as the measurement variable of the latent variable (or the factor) [27]. AMOS21.0 was performed to carry out confirmatory factor analysis on the fitting of two-factor model of SSFS. The expected value of *χ*^2^/df in various indicators is generally 2–3. To discuss the model fit of confirmatory factory analysis, it has been suggested that RMSEA value less than 0.05 is good, value between 0.08 and 0.1 is marginal, and value greater than 0.1 are poor [28]. Therefore, the RMSEA value of 0.04 in this sample indicates an acceptable fit. Indicators GFI and CFI > 0.90 represents acceptable fit of the model. Therefore, GFI of 0.945 and CFI of 0.967 indicate good fit. In summary, statistical analysis confirms SSFS to have high structural validity.

The discriminative validity indicates the degree to which a measured variable has weak or no correlation with other measured variables designed to measure other conceptual variables. This is usually performed by comparing the square root of the correlation coefficient and the square root of AVE between the variables [29]. In the scale, the postural assessment and the overall spinal function assessment had some degree of differentiation from each other, indicating that the scale had an ideal discriminative validity.

### Reliability analysis

Reliability analysis evaluates the magnitude of variance due to random errors during the assessment process [30]. This study incorporated the Cronbach’s *α* coefficient to evaluate the overall internal consistency of the scale, in other words, to examine whether the items of the scale measure the same construct [8, 11]. Streiner [31] suggested that the Cronbach *α* coefficient should not exceed 0.9 and that the acceptable standard is 0.4–0.5 [32]. The results of this study show that the SSFS has an alpha coefficient of 0.727, which indicates that the scale has high reliability. After removing each individual item, the *α* coefficients maintained between 0.692 and 0.717, implying high internal consistency between items of the scale.

Since this assessment scale was completed through self-assessment, inter-rater reliability analysis was not conducted. Instead, the Spearman correlation coefficient was used to evaluate the test-retest reliability, and the time internal for repeated administration of the scale was selected to be two weeks after the initial data collection [33]. Some researchers have also chosen a time interval of 24 h in functional evaluation studies of patients with severe muscle weakness [11], perhaps taken into account the influence of clinical progression on test-retest reliability. The results of this study showed an excellent test-retest reliability of the total score (*r* = 0.914, *P* < 0.01), as well as good test-retest reliability of the postural assessment factor (*r* = 0.800, *P* < 0.01) and of the functional assessment factor (*r* = 0.889, *P* < 0.01). For the individual items of the SSFS, other than supine postural assessment (*r* = 0.567, *P* < 0.01), prone press-up (*r* = 0.544, *P* < 0.01) and wall angel (*r* = 0.505, *P* < 0.01), other items resulted in good test-retest reliability (*r* > 0.6, *P* < 0.01). The reliability analysis determines the SSFS to have high reliability.

### Effect of BMI levels on the spinal function in healthy population

This study analyzed the effect of BMI levels on the SSFS score and examined 819 samples with complete information. These subjects include military recruits, postsecondary students, and regular workers. The rate of spinal dysfunction in the underweight group as well as overweight and obese group in both male and female subjects were significantly higher than that in the normal weight group (*P* < 0.05), indicating that excessive weight may increase the burden on the spine during movement, thus resulting in spinal dysfunction. For the purpose of the study, it has been subjectively determined that that a total score below 14 points indicates spinal dysfunction.

The results implied that the overall spinal function in the overweight and obese male subjects was significantly worse than that in the normal group (*P* < 0.05), suggesting that excessive weight in young men may increase the burden on the spine during exercise, resulting in dysfunction. The neck flexor strength test was significantly lower in the male underweight group than in the male normal group, indicating that the muscle mass of young men with lower BMI may be relatively lower, leading to poorer performance of muscle strength testing. In female subjects, the spinal function in the overweight and obese groups was lower than that in the normal group, with no significant difference (*P* > 0.05), which may be related to the smaller female sample size. In this study, the evaluation of lying postural assessment, neck flexor muscle strength, supine knee-to-chest, wall roll-down, and the total SSFS score of the overweight and obese female subjects were significantly lower than those of the underweight group (*P* < 0.05). The neck flexor muscle strength, prone press-back, wall angel, and total SSFS score of the normal group were significantly lower than those of the underweight group (*P* < 0.05). This suggests higher neck flexor muscle strength and better spinal posture and function in the underweight female subjects than in the overweight and normal weight female subjects. This finding may be related to the different exercise habits, physiological characteristics, and occupation in young healthy female population. Further investigation with larger sample size and in-depth objective analysis may be suggested.

### Inadequacies and the way forward

The sample population of this study is all young and healthy subjects; therefore, the statistical results of this study are only applicable to this particular population for the assessment and monitoring of spinal health and the prevention of related disorders. For patients with existing spine-related injuries, the reliability and validity of this scale still needs further research and verification, especially the influence of sex, age, occupation, and other factors on the scale scores. In addition, the proportion of female subjects was less than 18%, which may potentially lead to errors in the influence of gender differences on the scale score in this study. Therefore, further investigation and research efforts are recommended.

The selection of tests in SSFS adopted a combination of quantitative test and empirical evaluation, and subjects’ spinal function was categorized into three levels: good, satisfactory, and poor. The scientificity and accuracy still need to be further examined.

The utilization and completion of this scale set requirements on the cognitive, comprehensive, and operational capabilities of the subjects. Therefore, to improve the evaluation efficiency and enhance subject comprehension, the SSFS can be administered with supplementary diagrams, animations, or videos of the standardized test items in addition to the current text instructions. By integrating the engineering and material science methodology, the intelligent and accurate spine functional assessment and rehabilitation tools can be developed to further improve the reliability scale and the safety of the assessment process, which can then be applicable to a wider population in the general public.

## Conclusion

The novel Self-Reported Spine Functional Scale (SSFS) has high reliability and validity and can help to provide a reliable basis for the self-awareness and maintenance of spine functional conditions and prevent early onset of related spinal disorders in the young and healthy population.

Body weight has a significant influence on the SSFS score in healthy young adults. Overweight and obese male subjects are more likely to have spinal dysfunction, while underweight males displayed poor cervical flexor muscle strength. Underweight females were found to have better overall spinal function and stronger cervical flexor muscle strength.

## Supplementary Information

**Additional file 1.** Self-Reported Spine Functional Assessment Form.

**Additional file 2.** Self-Reported Spine Functional Scale (SSFS).

## Data Availability

The datasets used or analyzed during the current study are available from the corresponding author on reasonable request.

## Abbreviations

SSFS: Self-Reported Spine Functional Scale
AVE: Average variance extracted analysis
ODI: Oswestry Disability Index
NDI: Neck Disability Index
RMSEA: Root mean square error of approximation
GFI: Goodness-of-fit index
CFI: Comparative fitness index
BMI: Body mass index
