# Supplementary material for: Reliability and validity of the novel self-reported spine functional scale (SSFS) in healthy participants
Source: J Orthop Surg Res. 2021 Aug 25;16:529. doi: 10.1186/s13018-021-02620-1 (PMC8386065; doi:10.1186/s13018-021-02620-1)
Supplement: Supplementary file 1 — Additional file 1. Self-Reported Spine Functional Assessment Form. [file 13018_2021_2620_MOESM1_ESM.docx]

**Additional file 1**

**Self-Reported Spine Functional Assessment Form**

Name： Sex： Age： Ht (cm)： Wt (kg)： Occupation： Routine Exercise：Y**□** N**□**

|  | Items | | | **Score** | | | | Score |
| --- | --- | --- | --- | --- | --- | --- | --- | --- |
|  |  |  |  | 0 | 1 | 2 | 3 |  |
| Postural Assessment | Upright | Ear-to-Acromion Alignment | | All items are abnormal | 1 item is normal | 2-3 items are normal | All items are normal |  |
|  |  | Shoulder Level Symmetry | |  |  |  |  |  |
|  |  | Levels of ASIS | |  |  |  |  |  |
|  |  | Lumbar Spine Curvature | |  |  |  |  |  |
|  | Recumbent | Dorsal | Acromion Levels | All items are abnormal | 1 item is normal | 2 items are normal | All items are normal |  |
|  |  |  | Nasal Line |  |  |  |  |  |
|  |  | Lateral | Lumbar Curvature |  |  |  |  |  |
| Muscle Strength | Neck Flexor Muscles | | | Subject cannot lift head off the surface, or compensatory movement (e.g. chin lift) was observed during effort | Subject is able to lift head off the surface, but cannot withstand external exertion of two-finger resistance applied to the forehead | Subject is able to lift head off the surface against two-finger resistance for two seconds and but is unable to withstand one-palm resistance | Subject is able to lift head off the surface against one-palm resistance applied to the forehead and hold the position for more than 2 seconds |  |
|  | Abdominal Core Muscles | | | Subject cannot hold the plank position supported on elbows and feet for > 1 minute | Subject is able to hold the plank position supported on elbows and feet for > 1 minute | Subject is able to hold the plank position with elevated contralateral elbow and lower limb for > 15 seconds | Subject is able to hold the plank position with elevated contralateral palm and lower limb for > 15 seconds |  |
| Functional Assessment | Prone Press-up | | | Subject cannot extend back and lift the upper body with bilateral elbows; or if the position of ASIS is visibly lifted from the surface with more than two-finger width distance | Subject is able to extend back and lift the upper body with bilateral elbows, but not with bilateral hands; or if the position of ASIS is visibly lifted from the surface with more than two-finger width distance | Subject is able to extend back and lift the upper body with bilateral hands; however, subject cannot move hands towards pelvis, or if during movement, the position of ASIS is visibly lifted from the surface with more than two-finger width distance | Subject is able to extend back and support upper body on both palms and can move bilateral hands towards the pelvis for more than one palm distance while keeping ASIS in the starting position |  |
|  | Supine Knee-to-Chest | | | Subject cannot lift hips off the surface | Subject is able to lift hips off the surface however the PSIS cannot be lifted up from the surface | Subject is able to lift hips at the level of PSIS up from the surface, but the lower lumbar vertebrae still remain contact with the surface | Subject is able to lift hips and lower lumbar vertebrae off the surface and there is a visible upward curve in the pelvic region when viewed from the side |  |
|  | Wall Roll-Down | | | Subject cannot reach maximum neck forward flexion; or shoulders or upper back compensate by losing contact from the wall surface during neck forward flexion | Subject is able to reach maximum neck flexion; however, when the shoulders roll down from the wall, the upper back compensate by losing contact from the wall surface during forward flexion | Subject is able to reach maximum neck flexion, roll down the shoulders, and forward bend the upper back step-by-step; but the lumbopelvic region compensates by losing contact from the wall surface during forward flexion | Subject is able to reach maximum neck flexion, roll down the shoulders, and forward bend the upper back step-by-step, with the lumbar spine firmly pressed against the wall; and then subject is able to move the lumbar spine away from the wall from upper segments to the lower segments, until the subject’s hips lose contact from the wall surface or until subject cannot maintain static standing position |  |
|  | Wall Angel | | | Subject cannot keep bilateral upper limbs in contact with the wall surface at the same time | Subject is able to keep bilateral upper limbs in contact with the wall surface but cannot slide arms upward; or if any one or more parts of the elbows, wrists or the back of hands lose contact from the wall surface during sliding | Subject is able to keep bilateral upper limbs in contact with the wall surface during upward sliding, but limbs cannot be completely straightened; or if any one or more parts of the elbows, the wrists or the back of hands lose contact from the wall during straightening | Subject is able to slide bilateral upper limbs up the wall while maintaining contact with the wall surface until both arms are completely straightened |  |
| Total Score | Overall Spinal Function：Max Score = 24; Good：20 - 24；Satisfactory：15 - 19；Unsatisfactory：0 - 14  Special Spinal Function Assessment: Omitted | | | | | | |  |
