# Supplementary material for: Reliability and validity of the novel self-reported spine functional scale (SSFS) in healthy participants
Source: J Orthop Surg Res. 2021 Aug 25;16:529. doi: 10.1186/s13018-021-02620-1 (PMC8386065; doi:10.1186/s13018-021-02620-1)
Supplement: Supplementary file 2 — Additional file 2. Self-Reported Spine Functional Scale (SSFS). [file 13018_2021_2620_MOESM2_ESM.docx]

**Additional file 2**

**Self-Reported Spine Functional Scale (SSFS)**

Spine Postural Assessment in the Upright Position: the cervical spine in the sagittal plane, the thoracic spine in the coronal plane, the lumbar spine and pelvis in the coronal and sagittal planes are evaluated by four observational assessment components, respectively: the alignment between the ear and the acromion, shoulder level symmetry, levels of the anterior superior iliac spine (ASIS), and the lumbar spine curvature. A score of 0 indicates all items being abnormal; a score of 1 indicates 1 of 4 items being normal; a score of 2 indicates 2-3 of 4 items being normal; a score of 3 indicates all 4 items being normal. (Figure 1 – 4)


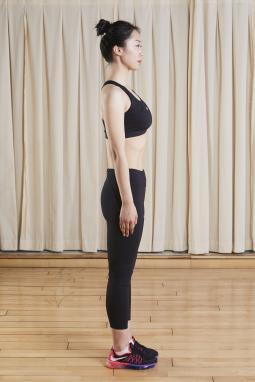

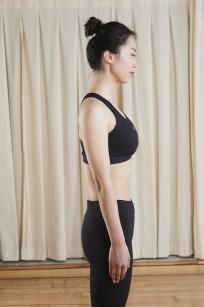

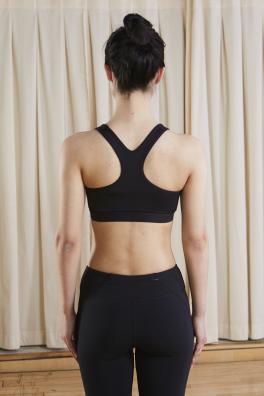

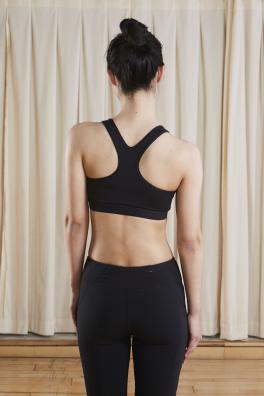


Normal Abnormal Normal Abnormal

Figure 1. Alignment between Acromion and Ear Figure 2. Shoulder Level Symmetry


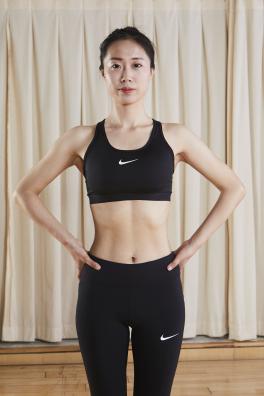

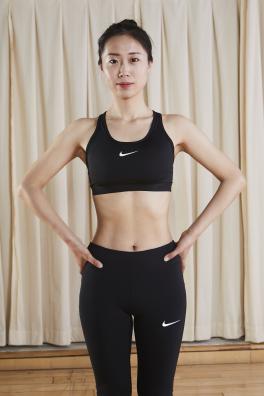

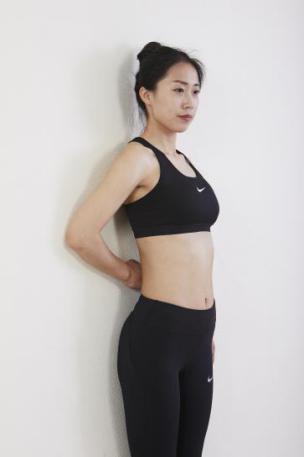

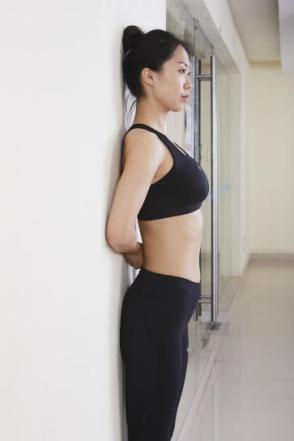

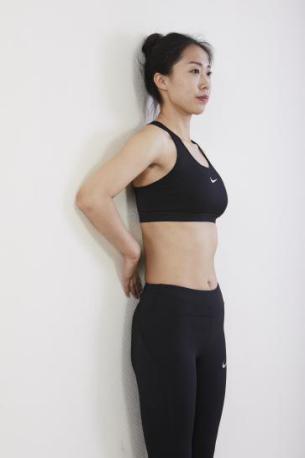


Normal Abnormal Normal Abnormal Abnormal

Figure 3. Levels of ASIS Figure 4. Lumbar Spine Curvature

Spine Postural Assessment in the Recumbent Position: the thoracic spine in the horizontal plane, three-dimensional orientation of the cervical spine, and the lumbar spine in the sagittal plane are evaluated using three observational assessment components, respectively: the level of the acromion, the position of the nasal line, and the side-lying lumbar curvature. A score of 0 indicates all items being abnormal; a score of 1 indicates 1 of 3 items being normal; a score of 2 indicates 2 of 3 items being normal; a score of 3 indicates all 3 items being normal. (Figure 5-7)


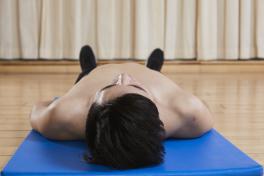

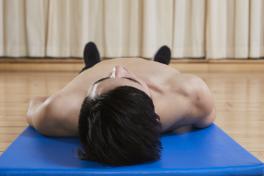

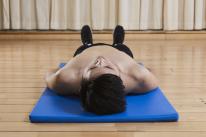

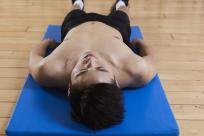


Normal Abnormal Normal Abnormal

Figure 5. Levels of Acromion Figure 6. Position of Nasal Line


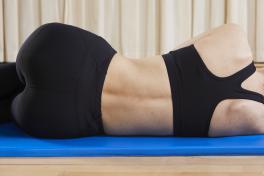

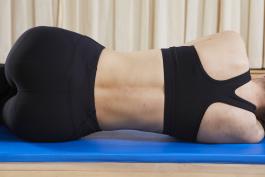


Normal Abnormal

Figure 7. Side-lying Lumbar Curvature

Neck Flexor Muscles Strength Testing: to assess the strength of cervical muscles when the subject actively flexes neck in the supine position. A score of 0 is allotted if the subject cannot lift head off the cushion surface, or if compensatory movement such as chin lift was observed during effort. A score of 1 is allotted if during chin-tuck neck flexion, the subject is able to lift head off the cushion surface, but cannot withstand external exertion of two-finger resistance applied to the forehead (i.e. if the subject’s chin lifts up or if subject cannot hold position for two seconds). A score of 2 is allotted if the subject is able to lift head off the surface against two-finger resistance for two seconds but is unable to withstand one-palm resistance (i.e. if chin lifts up to compensate or if subject cannot hold position for two seconds). A score of 3 points is allotted if the subject is able to lift head off the cushion surface against the one-palm resistance applied to the forehead and hold the position for more than 2 seconds. (Figure 8)


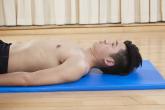

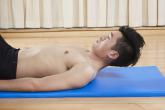

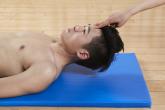

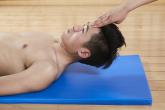


0 Point 1 Point 2 Points 3 Points

Figure 8 Neck Flexor Muscles Strength Testing Scoring Demonstration

Abdominal Core Muscles Strength Testing: to assess the spinal muscle strength, core stability, and motor coordination. The subject is asked to be in the plank position: face down, support trunk on bilateral elbows and feet. A score of 0 is allotted if the subject cannot hold the plank position for one minute. A score of 1 is allotted if the subject is able to hold the plank position for more than one minute. A score of 2 is allotted if the subject can elevate an upper limb and the contralateral lower limb while holding the plank position on one elbow and foot for more than 15 seconds. A score of 3 is allotted if subject can elevate the contralateral upper limb and the contralateral lower limb (opposite from the previous position), and maintain stable trunk position supported on one palm and one foot for more than 15 seconds. (Figure 9)


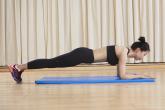

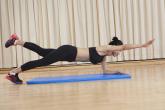

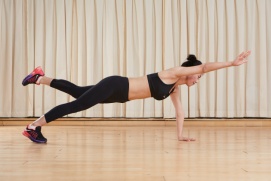


1 Point 2 Points 3 Points

Figure 9 Abdominal Core Muscles Strength Testing Scoring Demonstration

Prone Press-Up: to evaluate the dynamic posture and spinal movement in the sagittal plane and coronal plane, as well as to assess the coordination of spinal movements. A score of 0 is allotted if the subject cannot extend the back and lift the upper body with bilateral elbows, or if the position of the ASIS is visibly lifted from the cushion surface with more than two-finger width distance. A score of 1 is allotted if the subject is able to extend back and lift the upper body with bilateral elbows, but not with bilateral hands; or if the position of the ASIS is visibly lifted from the cushion surface with more than two-finger width distance. A score of 2 is allotted if the subject is able to extend back and lift the upper body with bilateral hands; however, the subject cannot move hands towards the pelvis, or if during movement, the position of the ASIS is visibly lifted from the surface with more than two-finger width distance. A score of 3 is allotted if the subject is able to extend back and support upper body on both palms and can move bilateral hands towards the pelvis for more than one palm distance while keeping ASIS in the starting position. (Figure 10)


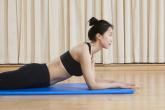

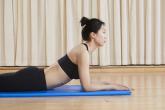

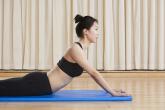

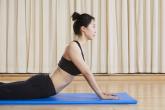


0 Point 1 Point 2 Points 3 Points

Figure 10 Prone Press-Up Assessment Scoring Demonstration

Supine Knee-to-Chest: to evaluate the dynamic posture and movement of the lumbosacral spine and pelvis in the sagittal plane, and spinal movement coordination. A score of 0 is allotted if the subject cannot lift hips (at the location where hip circumference is taken) off the cushion surface. A score of 1 is allotted if the subject is able to lift hips off the surface however the posterior superior iliac crest (PSIS) cannot be lifted up from the surface. A score of 2 is allotted if the subject is able to lift hips at the level of the PSIS up from the surface, but the lower lumbar vertebrae still remain contact with the cushion surface (there is no obvious upward curve in the pelvic region when viewed from the side). A score of 3 is allotted if the subject is able to lift hips and lumbar vertebrae off the cushion surface and there is a visible upward curve in the pelvic region when viewed from the side. (Figure 11)


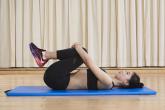

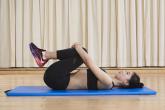

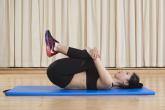

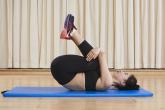


0 Point 1 Point 2 Points 3 Points

Figure 11 Supine Knee-to-Chest Assessment Scoring Demonstration

Wall Roll-Down: to evaluate the spinal posture and movement in the sagittal plane, the intersegmental mobility and stability; and the overall spinal movement coordination. A score of 0 is allotted if the subject cannot reach maximum neck forward flexion; or the subject’s shoulders as well as the areas below compensate by losing contact from the wall surface during neck forward flexion. A score of 1 is allotted if the subject is able to reach the maximum neck flexion; however, when the shoulders roll down from the wall, the upper back compensates by losing contact from the wall surface during forward flexion. A score of 2 is allotted if the subject is able to reach maximum neck flexion, roll down the shoulders, and forward bend the upper back in a step-by-step movement; but the lumbopelvic region compensates by losing contact from the wall surface during forward flexion. A score of 3 is allotted if the subject is able to reach maximum neck flexion, roll down the shoulders, and forward bend the upper back with the lumbar spine firmly pressed against the wall; and then the subject is able to move the lumbar spine away from the wall from upper segments to the lower segments, until the subject’s hips lose contact from the wall surface or until subject cannot maintain static standing position. (Picture 12)


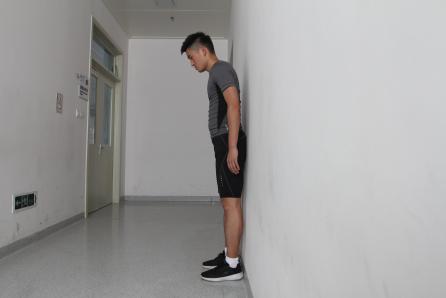

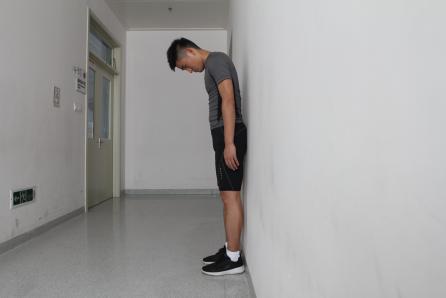

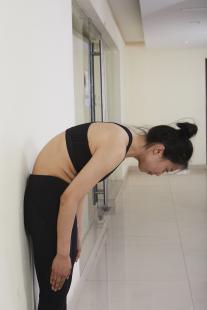

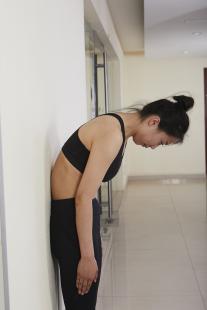


0 Point 1 Point 2 Points 3 Points

Figure 12 Wall Roll-Down Assessment Scoring Demonstration

Wall Angel: to evaluate the posture and mobility of the thoracic spine in the sagittal plane, the range of motion of the upper limbs, and spinal movement coordination. A score of 0 is allotted if the subject cannot keep bilateral upper limbs in contact with the wall surface at the same time. A score of 1 is allotted if the subject is able to keep bilateral upper limbs in contact with the wall surface but cannot slide arms upward; or if any one or more parts of the elbows, wrists or the back of hands lose contact from the wall surface during sliding. A score of 2 is allotted if the subject is able to keep bilateral upper limbs in contact with the wall surface during upward sliding, but arms cannot be completely straightened; or if any one or more parts of the elbows, the wrists or the back of hands lose contact from the wall during straightening. A score of 3 is allotted if the subject is able to slide bilateral upper limbs up the wall while maintaining contact with the wall surface until both arms are completely straightened. (Figure 13)


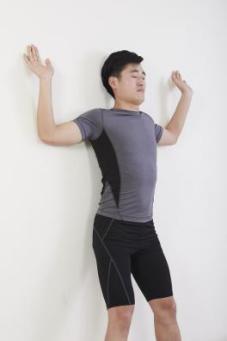

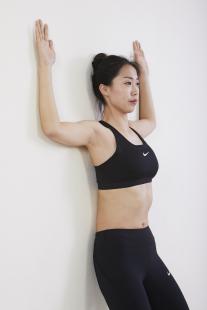

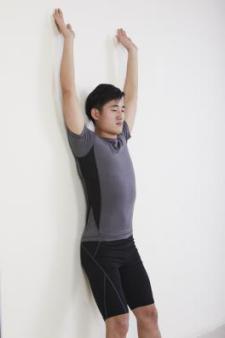

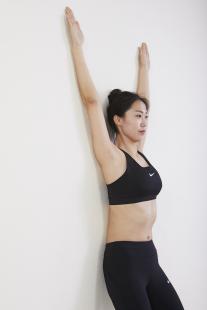


0 Point 1 Point 2 Points 3 Points

Figure 13 Wall Angel Assessment Scoring Demonstration
